# Zinc Status Biomarkers and Cardiometabolic Risk Factors in Metabolic Syndrome: A Case Control Study

**DOI:** 10.3390/nu9020175

**Published:** 2017-02-22

**Authors:** Erika P. S. Freitas, Aline T. O. Cunha, Sephora L. S. Aquino, Lucia F. C. Pedrosa, Severina C. V. C. Lima, Josivan G. Lima, Maria G. Almeida, Karine C. M. Sena-Evangelista

**Affiliations:** 1 Postgraduate Nutrition Program, Center for Health Sciences, Federal University of Rio Grande do Norte, Natal RN 59078-970, Brazil; erikapsf@hotmail.com (E.P.S.F.); sephoralouyse@msn.com (S.L.S.A.); lfcpedrosa@gmail.com (L.F.C.P.); scvclima@gmail.com (S.C.V.C.L.); 2 Postgraduate Program in Health Sciences, Center for Health Sciences, Federal University of Rio Grande do Norte, Natal RN 59012-570, Brazil; nine_tuane@hotmail.com; 3 Department of Nutrition, Center for Health Sciences, Federal University of Rio Grande do Norte, Natal RN 59078-970, Brazil; 4 Department of Clinical Medicine, Federal University of Rio Grande do Norte, Natal RN 59010-180, Brazil; josivanlima@gmail.com; 5 Department of Clinical and Toxicological Analysis, Federal University of Rio Grande do Norte, Natal RN 59012-570, Brazil; mgalmeida84@gmail.com

**Keywords:** metabolic syndrome, zinc, biomarkers, risk factors, cardiovascular diseases

## Abstract

Metabolic syndrome (MS) involves pathophysiological alterations that might compromise zinc status. The aim of this study was to evaluate zinc status biomarkers and their associations with cardiometabolic factors in patients with MS. Our case control study included 88 patients with MS and 37 controls. We performed clinical and anthropometric assessments and obtained lipid, glycemic, and inflammatory profiles. We also evaluated zinc intake, plasma zinc, erythrocyte zinc, and 24-h urinary zinc excretion. The average zinc intake was significantly lower in the MS group (*p* < 0.001). Regression models indicated no significant differences in plasma zinc concentration (all *p* > 0.05) between the two groups. We found significantly higher erythrocyte zinc concentration in the MS group (*p* < 0.001) independent from co-variable adjustments. Twenty-four hour urinary zinc excretion was significantly higher in the MS group (*p* = 0.008), and adjustments for age and sex explained 21% of the difference (*R*^2^ = 0.21, *p* < 0.001). There were significant associations between zincuria and fasting blood glucose concentration (*r* = 0.479), waist circumference (*r* = 0.253), triglyceride concentration (*r* = 0.360), glycated hemoglobin concentration (*r* = 0.250), homeostatic model assessment—insulin resistance (*r* = 0.223), and high-sensitivity C-reactive protein concentration (*r* = 0.427) (all *p* < 0.05) in the MS group. Patients with MS had alterations in zinc metabolism mainly characterized by an increase in erythrocyte zinc and higher zincuria.

## 1. Introduction

Metabolic syndrome (MS) is a multifactorial disease, and is influenced by metabolic, genetic, and environmental factors. The main MS alterations are glucose intolerance, abdominal adiposity, arterial hypertension, atherogenic dyslipidemia, and the consequent manifestations of pro-inflammatory and pro-thrombotic states [1]. The prevalence of MS and associated factors has been studied in many populations. In Latin-American countries, including Brazil, the prevalence is 24.9% [2].

Zinc has attracted interest in studies of its relations to MS due to the fact that this mineral has structural, catalytic, and regulatory roles in many proteins involved in pathophysiological mechanisms related to MS [3]. Zinc is involved in insulin synthesis and secretion [4,5] and blood pressure regulation [6], and has antioxidant and anti-inflammatory properties [7,8]. Inadequate zinc intake seems to lead to the occurrence of MS or its components [9,10]. However, studies evaluating zinc status in patients with MS have been controversial, and have had inconclusive results [11,12,13]. Some of these studies demonstrated an association of zinc with MS components, such as fasting blood glucose (FBG) concentration [14,15], blood pressure [13,16], and abdominal obesity [16,17]. Additionally, some authors have shown that greater numbers of MS components are associated with lower plasma zinc concentrations [13].

Considering the fact that there are no zinc stores in organisms, and that homeostatic control of zinc is complex [18], the search for a reliable indicator for zinc status has been problematic [19]. There are gaps in the literature regarding accurate assessment of zinc status, especially in mild to moderate deficiency, and the findings of the zinc biomarkers are conflicting and inconsistent [20]. Plasma zinc is currently the most widely used and accepted biomarker of zinc status, despite poor sensitivity and nonspecificity [19]. Some authors suggest the investigation of a more sensitive biomarker of zinc status for evaluation in the clinical management of patients with MS [20].

More recently, an international expert panel pointed to a 24-h urinary zinc excretion as a potential zinc biomarker, although some metabolic or physiologic conditions increase protein catabolism and, therefore increase urinary zinc [21]. However, there are not enough data on urinary zinc to support consistent recommendations on the validity and usefulness as an indicator for zinc status [22,23]. The regulation of cellular zinc is complex, and may involve both fast and slow mechanisms for regulating cellular zinc turnover [18], so cellular biomarkers such as erythrocyte zinc may lead to novel biomarkers of zinc status [21]. 

Therefore, investigating zinc status through an isolated marker can affect the conclusions of studies or generalizations made about specific results [24]. Thus, in order to better understand the relationship between zinc and MS, we evaluated zinc status biomarkers and their associations with cardiometabolic risk factors in individuals with MS.

## 2. Materials and Methods

### 2.1. Study Population

The inclusion criteria for the MS group were age of 20–76 years, both sexes, and the presence of MS according to the criteria from the National Cholesterol Education Program—Third Adult Treatment Panel (NCEP-ATPIII) [25], which includes the presence of at least three of the following alterations: waist circumference (WC) >102 cm in men and >88 cm in women; triglyceride (TG) concentration ≥150 mg/dL; high-density lipoprotein cholesterol (HDL-c) <40 mg/dL in men and <50 mg/dL in women; systolic blood pressure (SBP) ≥130 mmHg and/or diastolic blood pressure (DBP) ≥85 mmHg and/or use of antihypertensive drugs and FBG ≥100 mg/dL and/or use of oral antidiabetic drugs. The FBG cut-off point was adjusted according to guidelines from the American Diabetes Association [26]. The inclusion criteria for the control group were WC <102 cm in men and <88 cm in women, absence of arterial hypertension, diabetes mellitus (DM), insulin resistance, and the absence of antihypertensive and antidiabetic drug use.

The exclusion criteria for both groups were: type 1 DM, type 2 DM with insulin use, use of glucocorticoids in the last 3 months, use of vitamin-mineral supplements in the past 3 months, alterations in renal and hepatic function, and decompensated heart failure. Patients with memory or mental health issues and pregnant and nursing patients were also excluded.

The study population consisted of 88 patients with MS and 37 controls. Between March 2015 and February 2016, 875 medical records were screened, from which 119 individuals met the criteria for MS. However, 26 individuals did not appear for their clinical appointment, and five refused to be a part of the research, leading to a total of 88 patients. Two-hundred and fifty-three individuals were contacted to be a part of the control group, but only 37 met the specific inclusion criteria.

The study was conducted in accordance with the Declaration of Helsinki, and the protocol was approved by the Hospital Universitario Onofre Lopes’s Research Ethics Committee under protocol number CAAE No. 38566914.5.0000.5292, of 27 February 2015. All participants gave their informed consent for inclusion before they participated in the study. 

### 2.2. Study Design

**Figure 1:**
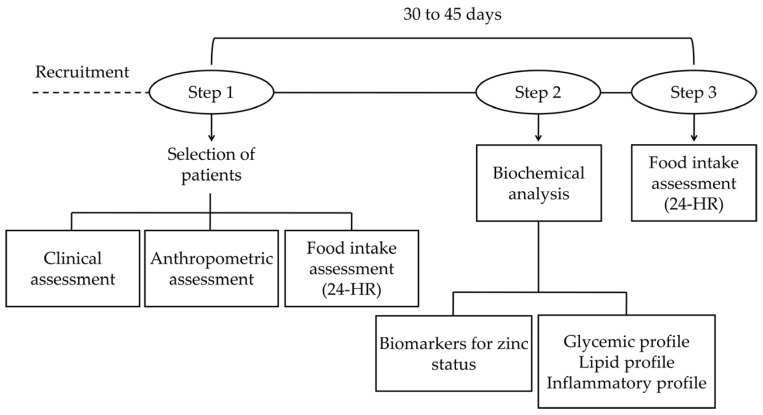
Study design.

The longitudinal study—which was a non-paired case–control study—was conducted at the Endocrinology Ambulatory department of Hospital Universitario Onofre Lopes, located in Natal/RN, Northeast Brazil. During the first appointment, the individual underwent a clinical assessment, where blood pressure was measured (mercury column blood pressure Plus device, SKU 001 model, Unitec^®^, São Paulo, SP, Brazil), as recommended by the Sixth Edition of the Brazilian Guidelines on Arterial Hypertension [27]. The subject’s weight (digital balance, MEA-03140 model, Tanita^®^, Arlington Heights, IL, USA) and stature (portable stadiometer personal caprice, ES2060 model, Sanny^®^, São Paulo, SP, Brazil) were measured for body mass index (BMI) calculation. The BMIs were classified according to the cut-off points recommended by the Brazilian Food and Nutrition Surveillance System [28]. We measured the WC at the midpoint between the last rib and the iliac crest, using an inextensible measuring tape. We also carried out a questionnaire on personal and health information. The individual’s food intake was assessed by 24-h recall at both time points, with an interval of 30–45 days. Blood and urine sample collection for biochemical analysis was scheduled at Step 2 (Figure 1).

### 2.3. Food Intake Assessment

Habitual food intake was investigated using two 24-HRs, with an interval of 30–45 days. The subject’s diet was analyzed using the Virtual Nutri Plus 2.0^®^ software (São Paulo, Brazil). Nutritional information for new preparations and foods were added to the database as necessary, along with data regarding nutritional composition from the Brazilian food composition table (TACO) and the United States Department of Agriculture (USDA).

The prevalence of inadequate zinc intake was estimated according to sex and age, using the estimated average requirements (EAR) cut-point method [29,30], considering the proportion of individuals with intake below the EAR value. Zinc values were adjusted based on intra- and inter-personal variability and energy [31,32].

### 2.4. Biochemical Tests

Blood samples (20 mL) were collected via venipuncture using disposable syringes after an overnight fasting of 12 h. Enzymatic method was used for FBG, total cholesterol, and TG analysis. HDL-c concentration was determined via a direct colorimetric test, and low density lipoprotein cholesterol (LDL-c) concentration was obtained using the Friedewald formula [33]. Non-HDL-c was calculated using the formula:

non-HDL-c = CT-HDL-c(1)

The high-sensitivity C-reactive protein (hs-CRP) analysis was assessed by immunoturbidimetric method with latex using CMD 800ix1 autoanalyze, and Turbitest AA Line Wiener Lab^®^ kits (Wiener lab group, Rosario, Argentina). Immunoturbidimetric inhibition assay was performed to quantify glycated hemoglobin (HbA1c) concentration also using CMD 800iX1 autoanalyze and Wiener Lab^®^ kits (Wiener lab group, Rosario, Argentina).

Insulin determination was performed by a chemiluminescent test, and an analysis kit from Beckman Coulter^®^ (immunoassay system, Unicel^®^ DxI800, Beckman Coulter, Brea, CA, USA). To evaluate insulin resistance, we used the homeostasis model assessment—insulin resistance (HOMA-IR) index, which was obtained using insulin fasting values and FBG:

HOMA-IR = [(insulin µU/mL × FBG mmol/dL)/22.5](2)

#### Zinc Status Assessment Biomarkers

Zinc status assessment was performed using plasma zinc, erythrocyte zinc, and 24-h urinary zinc excretion. To minimize mineral contamination, all glassware and plastic containers used during the blood collection and zinc analysis were carefully demineralized in a 20% nitric acid bath for at least 12 h and rinsed 10 times with ultra-pure water (Direct-Q^®^ 3 Water Purification Systems, Merck Millipore, Darmstadt, Germany).

Blood samples used for zinc analysis was placed in demineralized tubes containing 100 µL of 30% sodium citrate solution and separated into plasma and erythrocytes. In order to carry out the urinary zinc excretion analysis, we performed urine collection over a period of 24 h. Plasma zinc, erythrocyte zinc, and urinary zinc concentrations were determined by atomic absorption spectrophotometry, using Spectra Varian^®^AA-240 (Varian Medical Systems, Inc., Milpitas, CA, USA). We calibrated the assay using the following working conditions: wavelength, 231.9 nm; slit width, 1.0 nm; current, 5.0 mA; expansion factor, 1.0; and sample flow, 5 mL/minute. A standard zinc solution (Tritisol^®^, Merck, Darmstadt, Germany) was used to define the points of the calibration curve, which included concentrations of 0.0, 0.1, 0.2, 0.3, 0.5, and 1.0 µg/mL. The standard curve for the reading of plasma zinc concentration was prepared with the addition of 5% glycerol. Seronorm™ Trace Elements Serum L-1 solution (SERO AS, Billingstad, Norway) (Reference 201405, Lot 0903106) was used as reference for zinc analysis.

Plasma zinc concentration was determined according to the method described by Rodrigues et al. [34]. We used values of 70 to 110 µg Zn/dL as reference for plasma zinc concentration [35]. Zinc analysis in erythrocytes was performed according to Whitehouse et al. [36], following the standardization of the methodology proposed by Sena et al. [37]. The results were expressed as µg zinc/g of hemoglobin, with reference value of 40–44 µg Zn/g Hb [35]. In order to determine hemoglobin concentration in erythrocytes, we used reagent kits from Labtest Diagnóstica^®^ (Lagoa Santa, MG, Brazil) and a color reagent (RCOR CAT 43). We performed the experiments using spectrophotometry at 540 nm (Hach Company, model DR 5000, Düsseldorf, Germany). Determination of 24-h urinary zinc excretion was carried out using the method proposed by Kiilerich et al. [38]. The results were expressed as µg Zn/24 h, with reference values of 300 to 600 µg Zn/24 h [39].

### 2.5. Statistical Analysis

Because of the low recruitment rate (125 individuals, 88 with MS and 37 controls), our study had a statistical power of 20.5% to detect a plasma zinc concentration difference of 4 µg/dL (or more) between the groups.

We first performed multiple missing data imputation techniques [40] for the following variables: insulin, HbA1c, HOMA-IR, TG, LDL-c, hs-CRP, plasma zinc, erythrocyte zinc, and urine zinc. Continuous variables were expressed as mean ± standard deviation (SD), mean (confidence interval (CI) of 95%), or median (interquartile interval), as appropriate. Continuous variable distributions were visually evaluated using histograms. Due to the moderately symmetrical characteristic of most variables, we used parametric approaches following the Fagerland guidelines [41]. Counts and proportions were used to summarize binary variables. For intragroup comparisons (case vs. control), we used the Student’s *t*-test for independent samples and Fisher’s exact test to test differences among continuous variables and proportions, respectively. Differences between cases and controls are indicated by “∆”, where ∆ = (case group mean) − (group control mean). The “∆” difference was adjusted to co-variables through simple and multiple linear regression models. The correlations between MS cardiometabolic risk factors and zinc biomarkers were measured using the Pearson’s correlation coefficient test (r). For variables with imputed observations, Pearson’s coefficient was computed using a simple linear regression coefficient. Statistical significance α was set at the 5% level. We used Stata 14.0 software (Stata Corporation, College Station, TX, USA).

## 3. Results

The general characteristics of the patients are summarized in Table 1. The mean age of patients with MS was significantly higher than that of subjects in the control group (50 (11) vs. 44 (11), *p* = 0.011). Females were predominant in both groups. The mean WC of patients with MS indicated an increased risk of cardiovascular disease. Patients with MS had dyslipidemia (92%), arterial hypertension (76%), and obesity (66%). We observed that 86% of the patients with MS met more than three MS diagnosis criteria. The most frequent components found were arterial hypertension or altered blood pressure (90%), increased WC (86%), and altered FBG or type 2 DM (without insulin use) (76%).

We observed that the mean values of FBG, insulin, HbA1c (all *p* < 0.001), and HOMA-IR (*p* = 0.002) were significantly higher in the MS group than in the control group. Significantly lower values for HDL-c (*p* < 0.001) and higher TG values (*p* = 0.034) were observed in the MS group. The MS group had an inflammatory profile, as indicated by the significantly higher hs-CRP concentration (*p* < 0.001).

The MS group had significantly lower zinc intake compared to the control group (6.57 (1.64) mg/day vs. 9.37 (2.41) mg/day, *p* < 0.001). We found a high prevalence of inadequate zinc intake in the participants of this study (56% of women and 95% of men in the MS group and 18% of women and 40% of men in the control group). We did not observe statistically significant differences in plasma zinc concentration between groups (*p* > 0.05). However, erythrocyte zinc concentration in patients with MS were significantly higher compared to the control group (*p* < 0.001). Patients with MS had increased 24-h urinary zinc excretion compared to control subjects, and the difference was statistically significant (*p* = 0.008).

**Figure 2:**
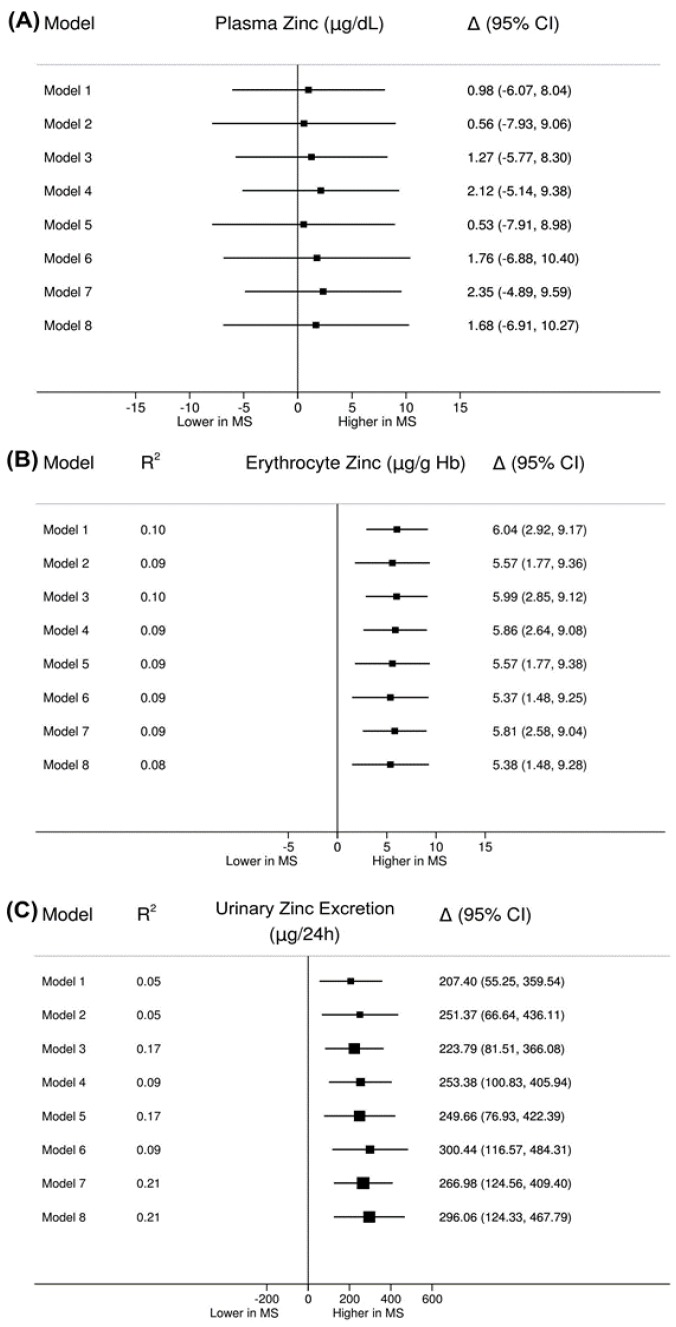
(**A**) Forest plot for comparison of plasma zinc concentration; (**B**) erythrocytes zinc concentration; and (**C**) 24-h urinary zinc excretion between patients with MS and the control group, according to the adjustment models. Model 1: without adjustment; Model 2: adjusted for zinc intake; Model 3: adjusted for sex; Model 4: adjusted for age; Model 5: Adjusted for zinc intake and sex; Model 6: Adjusted for zinc intake and age; Model 7: adjusted for sex and age; and Model 8: Adjusted for zinc intake, sex, and age. Horizontal lines indicate the 95% confidence interval (CI) for each model. When the confidence interval does not cross the vertical axis (centered at zero), the difference in the zinc biomarker concentration among the study groups is statistically significant (*p* < 0.05). If the confidence interval line is to the right of the central axis, it indicates that the zinc biomarker concentration is higher for patients with MS compared to the control group. In contrast, if it is on the left of the central axis, it indicates that the concentration is lower in the MS group in relation to the controls. R2: determination coefficient; ∆: estimates of the difference between patients with MS and controls, whose mean is inside the square (■); the area of each square is proportional to the variability explained by the model.

Figure 2 shows the multiple linear regression models created to evaluate zinc intake and sex and age influences on the differences in plasma (2A), erythrocyte (2B), and urine zinc (2C) between patients with MS and controls. The regression models confirmed that there are no statistically significant differences in plasma zinc concentration between patients with MS and controls independent of adjustment variables (all *p* > 0.05) (Figure 2A). Erythrocyte zinc concentration was significantly higher in patients with MS in all adjustment models (all *p* < 0.05). We observed that the model without adjustment (model 1) already explains 10% of the variability in this measure (R2 = 0.10, *p* < 0.001), confirming that there is no influence of co-variables, such as zinc intake, sex, and age on this measure (Figure 2B).

There were differences in 24-h urinary zinc excretion between the MS and control groups after adjustments for zinc intake, sex, age, and other combinations of variables (all *p* < 0.05). The MS group had a mean 24-h urinary zinc excretion 207.40 units higher than that of the control group (model 1). This difference increased following changes to the adjustment variables. The adjustment models 7 (sex and age) and 8 (zinc intake, sex, and age) explained the variability the most, with both explaining 21% of the variability (R^2^ = 0.21, *p* < 0.001) (Figure 2C). These models indicate that the mean 24-h urinary zinc excretion rate is 266.98 µg/24 h higher in the patients with MS group compared to controls.

We did not observe significant associations between the biomarkers of zinc status or zinc intake, plasma, and erythrocyte zinc concentration with cardiometabolic risk factors in any of the groups. We observed statistically significant positive correlations between 24-h urinary zinc excretion and WC (*r* = 0.253, *p* = 0.02), TG (*r* = 0.360, *p* < 0.01), FBG (*r* = 0.479, *p* < 0.01), HbA1c (*r* = 0.250, *p* = 0.02), HOMA-IR (*r* = 0.223, *p* = 0.04), and hs-CRP (*r* = 0.427, *p* < 0.01) in patients with MS. This indicates that zincuria increases are proportional to the increases in alterations of most of the MS components and other cardiometabolic risk factors. The same results were not found in the control group, with the exception of WC (*r* = 0.434, *p* < 0.01) and DBP (*r* = 0.499, *p* < 0.01) (Table 2).

## 4. Discussion

To our knowledge, this is the first study to evaluate zinc status in patients with MS, considering zinc intake, plasma zinc, erythrocyte zinc, and 24-h urinary zinc excretion. We also studied the associations between these biomarkers and cardiometabolic risk factors. We found plasma zinc concentration without any obvious indicative deficiencies in the MS group, although those patients had high prevalence of inadequate zinc intake. On the other hand, we observed increases in erythrocyte zinc concentration and 24-h urinary zinc excretion. Alterations in cardiometabolic risk factors were only positively associated with 24-h urinary zinc excretion in patients with MS.

Assessments of zinc intake and mineral bioavailability are considered to be the best methods for estimating zinc concentration at individual and population levels [21]. In our study, considering only dietary zinc intake, we suggest that patients with MS are at risk of zinc deficiency. Similarly, Cunha et al. [42] found a high percentage of zinc intake inadequacy in patients with MS, and especially in men (75.5%). Other authors have also observed mineral intake inadequacies and have identified inverse associations with MS risk [9,10]. A possible explanation for the zinc intake inadequacies in our study is the restriction placed on certain food sources—especially foods from animal origin—that is recommended as a part of the MS treatment. Although we did not find associations between dietary zinc intake and cardiometabolic factors in patients with MS, some authors have shown associations between deficiency in zinc intake and MS components, such as peripheral resistance to insulin [4], high blood pressure [6], high TG concentration and low HDL-c concentration [43], and central obesity [44].

One specific result that attracted our attention was the fact that we did not observe correlations between zinc intake and plasma zinc concentration. Additionally, we did not observe significant differences in plasma zinc concentration between patients with MS and controls. Only 15% of the participants had plasma zinc values below 70 µg/dL in either group. The literature is not very clear in this regard, as studies evaluating plasma zinc concentration in patients with MS are inconclusive. A study performed using 1926 Korean adults from both sexes also did not find significant differences in serum zinc concentration between individuals with and without MS [13]. In contrast to our findings, Rotter et al. [12] observed significantly higher serum zinc concentration in patients with MS than in controls in a study performed with 313 Polish men. In addition, in a populational study of 2,041 adult Iranians, it was found that higher serum zinc concentration was associated with a lower MS prevalence in female subjects, while the opposite result was observed in male subjects [16].

Despite only 1% of total zinc in the body being present in circulating blood, plasma zinc is an important biomarker, and is sensitive enough to detect serious deficiencies, but has limitations in identifying marginal deficiencies. However, in order to interpret zinc plasma concentration in clinical populations, other deficiency risk factors should be considered, such as inadequate dietary intake of zinc [21]. Similar to zinc intake, we did not observe correlations between plasma zinc and erythrocyte zinc concentrations, nor 24-h urinary zinc excretion. It is noteworthy that zinc has rigid homoeostatic control and easily moves among cell compartments in order to keep the plasma mineral concentration within an optimal range [18]. However, we have also observed satisfactory plasma zinc concentration, which may occur in order to avoid an impairment of homeostatic control [45]. Therefore, values within the reference range observed in patients with MS might not accurately reflect the total corporeal zinc, as zinc plasma concentration has fluctuations of around 20% during the day. Alterations in the metabolism of zinc may also occur following acute infection, hypoalbuminemia, and inflammation [19].

Some possible mechanisms may explain the plasma zinc concentration observed in our study: (1) zinc mobilization among cell compartments, plasma, the liver, and the bone [46]; (2) the excretion of this mineral by the gastrointestinal tract, as zinc excretion via the intestinal lumen might be decreased, which would then reduce endogenous losses through feces [21]; and (3) hyperglycemia, which is common in patients with MS and may stimulate insulin secretion, where part of the zinc pool found in beta cells is secreted into the plasma together with the hormone [4].

Although other authors have observed negative associations between serum zinc concentration and cardiometabolic risk factors, such as FBG, HOMA-IR, insulin, and blood pressure [13], and a positive association between serum zinc and HDL-c [16], we did not identify such associations in our study. This may be due to reduced numbers of participants, which resulted in a low statistical power for detecting outcomes of the plasma zinc variable. The rigid homeostatic control of the mineral implies that there are very small differences in plasma zinc concentration between cases and controls. Thus, to confirm such associations, higher numbers of participants will be necessary. Therefore, consistent with our inconclusive results regarding zinc status in the clinical population, some authors suggest that cell biomarkers might be recommended as new targets, and together with plasma zinc, provide more reliable information regarding zinc status in individuals [21]. Thus, we included the erythrocyte zinc assessment to fill the literature gaps regarding the use of this biomarker in patients with MS.

We found significantly higher erythrocyte zinc concentration in the MS group compared to the control group, regardless of the participants’ dietary zinc intake, sex, or age. The fact that we found no correlations between erythrocyte zinc concentration and cardiometabolic risk factors such as hs-CRP may be explained by the lack of responsiveness of erythrocyte zinc biomarker to variations in zinc intake [21]. However, the high erythrocyte zinc concentration observed in 64% of patients with MS supports a previous study by Dias et al. [47]. The authors of that study observed that patients with altered inflammatory profiles, similar to our patients, had mean erythrocyte zinc concentration above the reference values. By contrast, in obese women, mean erythrocyte zinc values were found to be below 40 µg/gHb, which was significantly lower than that observed in the control group [17].

Our results indicate that patients with MS have an inflammatory profile, which was confirmed by the high hs-CRP values, significantly higher than those of controls. Alterations in lipid profiles, obesity, and type 2 DM lead to inflammation and oxidative stress [48]. The presence of inflammatory markers leads to alterations in the regulation of the genes for transporters of zinc and metallothionein, which compromises the redistribution of minerals in blood cells [49]. It is suggested that oxidative stress is present in type 2 DM [50] and obesity [51]—which are frequent clinical conditions in our population—favors the liberation of metallothionein and increases intracellular zinc concentrations. Furthermore, oxidative damage induced by type 2 DM seems to be more prominent in erythrocytes. This favors increases in antioxidant concentrations as a mechanism of compensation to protect cells [50].

The increased 24-h urinary zinc excretion in patients with MS was the most remarkable alteration, although we observed normal plasma zinc concentration. We found that 30% of the patients had hyperzincuria. In agreement with our findings, Martins et al. [51] observed that 24-h urinary zinc excretion was significantly higher in obese women than in controls. In our study, the differences between the MS and control groups were better explained when we applied corrections for age and sex. In fact, increases in age lead to decreased renal efficiency, which contributes to the increase in 24-h urinary zinc excretion. In addition, differences in body composition between men and women explain the higher excretion of zinc in males [52]. This information reinforces the importance of corrections for these co-variables when considering zinc excretion.

The normal plasma zinc concentration observed in patients with MS in our study suggests that the zinc status may not have been necessarily compromised by an increase 24-h urinary zinc excretion. Studies in animals showed that hormonal changes induced by insulin and glucagon increased urinary zinc without changing plasma zinc concentrations [53]. These findings suggest that plasma zinc probably sustained an optimal range by a combination of the mechanisms described above. 

Our findings confirmed the strong influence of cardiometabolic risk factors on zincuria. The effects were especially significant for WC, hs-CRP, TG, and glycemic profile variables, regardless of dietary zinc intake. In a study of Chinese adults, it was found that DM or pre-DM subjects have higher zinc excretion [54]. Additionally, Feng et al. [55] found that urine zinc is positively correlated with FBG, and that this condition raises DM risk.

Certainly, MS cardiometabolic risk factors seem to have a more determinant role on zincuria than dietary zinc intake. In fact, the literature indicates that decreases in 24-h urinary zinc excretion are only observed with diets containing less than 1 mg zinc/day [21]. The association between cardiometabolic risk factors and zincuria (confirmed in our study) highlights the fact that this condition seems to result from alterations in glucose metabolism caused by MS pathophysiological mechanisms. Abdominal adiposity—which is a remarkable characteristic of patients with MS—stimulates an increase in the liberation of free fatty acids and results in an increase in hepatic TG synthesis. This mechanism leads to muscular and hepatic insulin resistance. Additionally, abdominal adiposity induces an increase in circular inflammatory mediators, which lead to hyperglycemia [1]. The glycosuria resulting from chronic hyperglycemia influences the active transport of zinc in renal tubular cells and promotes the increase in urinary loss of this mineral [55]. In addition, albumin and other enzymes can attach to zinc, and when excreted, can lead to excretion of this mineral [56].

Another factor that might explain the increase in 24-h urinary zinc excretion is the use of drugs for arterial hypertension, which was present in over 50% of our patients with MS. The results of a systematic review [57] confirmed that diuretics, angiotensin converting enzyme inhibitors, and angiotensin 2 receptor antagonists are related to increases in zinc urinary loss, which affects zinc metabolism.

Our work is not free from limitations. These limitations are as follows: (1) the study has a non-paired case–control design, but we used regression models to consider confounding variables such as age and sex, in addition to searching for individuals with similar social-economic conditions; (2) even before using several recruiting strategies, the reduced numbers of participants limited the statistical power of the analysis to detect differences in the primary variable—plasma zinc—between the two groups; (3) systematic and random mistakes in dietary intake evaluation are possible. However, the data were collected by trained professionals, who were careful to ensure the best reliability for information regarding zinc content in the tables used to track food composition.

## 5. Conclusions

Patients with MS had high prevalence of inadequate zinc intake and alterations in zinc metabolism, mainly characterized by an increase in erythrocyte zinc and higher 24-h urinary zinc excretion. Alterations in cardiometabolic components lead to higher zincuria in patients with MS. The results of this study highlight the importance of considering several biomarkers when assessing zinc status in MS and recognizing the relationship between this mineral and the pathophysiology of the disease. In addition, investigating biomarkers little explored in the literature has expanded our knowledge and provided us with more information regarding zinc status. MS is a multifactorial disease which each individual has his or her own characteristics, depending on a number of components involved in disease diagnosis. Thus, studying the phenotype of each individual as it relates to zinc biomarkers will provide more reliable answers regarding the role of zinc in the mechanisms involved in MS.

## Figures and Tables

**Table 1:** General characteristics, metabolic profiles, and zinc biomarkers. MS: metabolic syndrome.

Variables	Patients with MS (*n* = 88)	Control (*n* = 37)	*p*-Value
Age (year) ^a^	50 (11)	44 (11)	0.011
Sex (F/M)	64/24	25/12	0.666
WC (cm) ^a^	105.50 (12.60)	82.40 (6.98)	<0.001
Type 2 DM ^b^	43 (49)	0	<0.001
Arterial hypertension ^b^	67 (76)	0	<0.001
Obesity ^b^	58 (66)	0	<0.001
Dyslipidemia ^b^	81 (92)	23 (62)	<0.001
SBP (mmHg) ^a^	134.85 (18.28)	114.46 (7.24)	<0.001
DBP (mmHg) ^a^	88.47 (11.51)	74.59 (7.3)	<0.001
FBG (mg/dL) ^a^	119.70 (47.7)	84.70 (11.27)	<0.001
Insulin (µUI/mL) ^c^	8.87 (5.10–13.76)	4.95 (3.48–6.93)	<0.001
HbA1c (%) ^a^	6.91 (1.67)	5.40 (0.52)	<0.001
HOMA-IR ^c^	2.35 (0.94–3.55)	0.99 (0.73–1.42)	0.002
Total cholesterol (mg/dL) ^a^	210.73 (60.22)	212.97(49.08)	0.841
LDL-c (mg/dL) ^a^	128.28 (48.44)	134.70 (43.83)	0.488
HDL-c (mg/dL) ^a^	44.95 (9.32)	53.92 (11.34)	<0.001
TG (mg/dL) ^c^	156.00 (126.90–215.50)	106.00 (75.00–177.00)	0.034
Non HDL-c (mg/dL) ^a^	165.76 (57.99)	159.05 (48.76)	0.538
hs-CRP (mg/L) ^c^	1.98 (0.67–6.59)	0.34 (0.09–0.98)	<0.001
Zinc intake (mg/d) ^a^	6.57(1.64)	9.37(2.41)	<0.001
Plasma zinc (µg/dL) ^a^	88.81 (18.28)	87.82 (17.44)	0.783
Erythrocyte zinc (µg/g Hb) ^a^	47.47 (8.29)	41.43 (7.37)	<0.001
24-h urinary zinc excretion (µg/24 h) ^c^	554.80 (291.00–787.60)	375.40 (197.60–597.50)	0.008

^a^ Data presented as mean (standard deviation); ^b^ Data presented as *n* (%); ^c^ Data presented median (interquartile interval) WC, waist circumference; DM, diabetes mellitus; SBP, systolic blood pressure; DBP, diastolic blood pressure; FBG, fasting blood glucose; HbA1c, glycated hemoglobin; HOMA-IR, homeostasis model assessment—insulin resistance; LDL-c, low-density cholesterol; HDL-c, high-density lipoprotein cholesterol; TG, triglyceride; non HDL-c, non-high-density lipoprotein cholesterol; hs-CRP, high-sensitivity C-reactive protein.

**Table 2:** Correlations between 24-h urinary zinc excretion and cardiometabolic risk factors.

Variables	Patients with MS		Controls	
	*r*	*p*	*r*	*p*
WC (cm)	0.253	0.018	0.434	0.007
SBP (mmHg)	0.021	0.816	0.060	0.723
DBP (mmHg)	−0.048	0.676	0.488	0.002
TG (mg/dL)	0.360	<0.001	0.075	0.657
HDL-c (mg/dL)	−0.027	0.842	−0.248	0.138
FBG (mg/dL)	0.479	<0.001	0.147	0.384
HbA1c (%)	0.250	0.024	−0.142	0.403
Insulin (µUI/mL)	0.029	0.787	−0.091	0.575
HOMA-IR	0.223	0.041	−0.065	0.700
hs-CRP (mg/L)	0.427	<0.001	0.303	0.082

WC, waist circumference; SBP, systolic blood pressure; DBP, diastolic blood pressure; TG, triglyceride; HDL-c, high-density lipoprotein cholesterol; FBG, fasting blood glucose; HbA1c, glycated hemoglobin; HOMA-IR, homeostasis model assessment—insulin resistance; hs-CRP, high-sensitivity C-reactive protein.
